# Research and development for the malaria elimination/eradication roadmap

**DOI:** 10.1186/1475-2875-9-S2-I11

**Published:** 2010-10-20

**Authors:** Marcel Tanner, Pedro L Alonso

**Affiliations:** 1 Swiss Tropical and Public Health Institute, 4002 Basel, Switzerland; 2 University of Basel, 4003, Basel, Switzerland; 3 CRESIB-Hospital Clinic, University of Barcelona, Barcelona, Spain & Centro de Investigaçao em Saude da Manhiça, Mozambique

## 

The Roll Back Malaria/World Health Organization Global Malaria Action Plan sets out a plan for improved control, leading to regional elimination and ultimately aims to eradicate malaria. This goal constitutes a moral, humanitarian and economic imperative and the question is therefore not whether mankind should pursue the goal of malaria eradication, but rather when and how. Over the last decade, resources and control efforts have intensified to a level not seen since the early days of the eradication campaign in the late 1950s. Nonetheless, there is - according to WHO - ongoing malaria transmission in 108 countries, out of which 61 are now focusing on sustained control, 27 countries have embarked on elimination, and some are already being certified malaria free.

However, the ambitious goal of malaria eradication cannot be achieved with the tools and approaches currently available. Research and development of new tools must accompany and complement the Global Action Plan. We report about the process to establish the respective R&D agenda and about the key R&D issues where renewed attention and investments are required [1]:

• strengthened focus on *P.vivax: in vitro* culture and study of hypnozoites

• drugs to be used for mass drug administration to clear infections and provide prophylaxis to prevent new infections

• vaccines that aim at interrupting transmission

• new vector control approaches for (i) outdoor biting/resting mosquitoes and (ii) achieving permanent reductions of vectorial capacity in areas where transmission is predominantly due to A. *gambiae* new approaches for fast and accurate assessment of transmission at community level and strengthened diagnostic, monitoring, and surveillance tools/approaches that are linked and embedded in the health and social health systems

• tool kits to scientifically assess and determine health system readiness for moving from control to elimination efforts

• new approaches in mathematical modelling to inform target product profiles of tools, and predict expected outcomes of intervention strategies.

The focus lies not on single technologies or approaches, but takes into account the heterogeneities of the current malaria situation, the key to effectively complement the Global Action Plan and reach the final goal.
